# Fuel Cell Using Squid Axon Electrolyte and Its Proton Conductivity

**DOI:** 10.3390/jfb11040086

**Published:** 2020-12-03

**Authors:** Tomoki Furuseki, Yasumitsu Matsuo

**Affiliations:** Department of Life Science, Faculty of Science & Engineering, Setsunan University, Ikeda-Nakamachi, Neyagawa, Osaka 572-8508, Japan; ymatsuo@lif.setsunan.ac.jp

**Keywords:** fuel cell, ion channel, electrolyte, squid axon, proton conductivity, biomaterials

## Abstract

Fuel cells using biomaterials have the potential for environmentally friendly clean energy and have attracted a lot of interest. Moreover, biomaterials are expected to develop into in vivo electrical devices such as pacemakers with no side effects. Ion channels, which are membrane proteins, are known to have a fast ion transport capacity. Therefore, by using ion channels, the realization of fuel cell electrolytes with high-proton conductivity can be expected. In this study, we have fabricated a fuel cell using an ion channel electrolyte for the first time and investigated the electrical properties of the ion channel electrolyte. It was found that the fuel cell using the ion channel membrane shows a power density of 0.78 W/cm^2^ in the humidified condition. On the other hand, the power density of the fuel cell blocking the ion channel with the channel blocker drastically decreased. These results indicate that the fuel cell using the ion channel electrolyte operates through the existence of the ion channel and that the ion channel membrane can be used as the electrolyte of the fuel cell in humidified conditions. Furthermore, the proton conductivity of the ion channel electrolyte drastically increases above 85% relative humidity (RH) and becomes 2 × 10^−2^ S/m at 96% RH. This result indicates that the ion channel becomes active above 96%RH. In addition, it was deduced from the impedance analysis that the high proton conductivity of the ion channel electrolyte above 96% RH is caused by the activation of ion channels, which are closely related to the fractionalization of water molecule clusters. From these results, it was found that a fuel cell using the squid axon becomes a new fuel cell using the function of the ion channel above 96% RH.

## 1. Introduction

Fuel cells, which are known as clean energy, are focused on as next-generation environmentally friendly energy because fuel cells can obtain energy with high efficiency by utilizing the simple reaction of producing water from hydrogen and oxygen. A lot of investigations concerning highly proton-conductive electrolytes and low-cost catalysts for fuel cells are currently being carried out. Especially for fuel cell electrolytes, the development of low-cost electrolytes with high proton conductivity is strongly desired. It is well-known that biomaterials are abundant in nature, low cost, and environmentally friendly materials [1]. Recently, in the fields of medicine and pharmacy, research using biological membrane materials with embedded functional membrane proteins has been attracting attention [2,3]. Since the biological membrane is a natural material, it is environmentally friendly and has a safe effect on the human body. Recent research has attempted to use fuel cells as the power source for machines used in the human body, such as the development of pacemakers that use fuel cells as the power source [4]. Biological membranes possessing a high affinity for the human body are desired for machines used in the human body. Therefore, this research, which treats biological membranes as electrolytes for fuel cells, will be useful for developing devices such as pacemakers that can be incorporated into the body. Ion channels, which are some of the proteins, exhibit high ionic transport through passive transport using a concentration gradient [5,6,7,8,9,10,11,12,13]. Ion channels exist in many cell membranes from bacteria to mammals and are deeply involved in maintaining important biological functions. The existence of ion channels was suggested by Hodgkin and Huxley et al. using giant squid axons [14,15,16]. Later, the patch-clamp technique developed by Neher and Sakmann proved the existence of ion channels by measuring the current of a single ion channel [17]. Moreover, Doyle et al. reported the three-dimensional structure of ion channels by X-ray structural analysis [6]. In this way, studies on ion channels have been actively carried out in the fields of medicine and pharmacy, and the relation between the crystal structure and the effect concerning medicines has been investigated [2,3,4,5,6,7,8,9,10,17,18,19,20,21,22,23,24]. However, there is no research on using ion channels in the energy field. Recently, we have focused on biomaterials as a substitute material for conventional electrolytes and have exhibited that DNA, collagen, chitin, and chitosan, which are tissue-derived biomaterials, show proton conductivity and could become the electrolyte of fuel cells [24,25,26,27,28,29]. However, the value of proton conductivity in these materials is not high; therefore, the investigation of biomaterials with higher proton conductivity is desired. The ion channel, which is one of the biofunctions, has the potential for the realization of high-proton conductivity [30,31,32,33,34]. That is, higher proton conductivity can be expected to be realized using an ion channel having a high function as an electrolyte of a fuel cell. However, it is currently difficult to artificially prepare an ion channel with the stability and mechanical strength needed as a battery. It is also known that axons are important biological organs involved in neurotransmission in the living body, and various membrane proteins, including ion channels, exist. Many ion channels are concentrated in axons for neurotransmission. In the ion channel research, squid axon has been used for a long time [35,36,37,38,39,40,41] and it is known that the ion channels of squid axons have low ion selectivity and the channel gate is open at zero potential [35,36]. Furthermore, electrochemical studies of ion channels using squid axons are often conducted, and if the specimen size is sufficient, stable results can be obtained without being significantly affected by individual differences [35,36,37]. Therefore, in the present study, we have selected the squid axon as the ion channel membrane and have fabricated a fuel cell using the squid axon as the electrolyte membrane. In addition, the properties of proton conductivity in the ion channel membrane have been investigated. This study of a fuel cell electrolyte using the ion channel will be helpful for the development of new fuel cell electrolytes.

## 2. Materials and Methods

### 2.1. Sample Preparation and Fabrication of Fuel Cell

**Figure 1:**
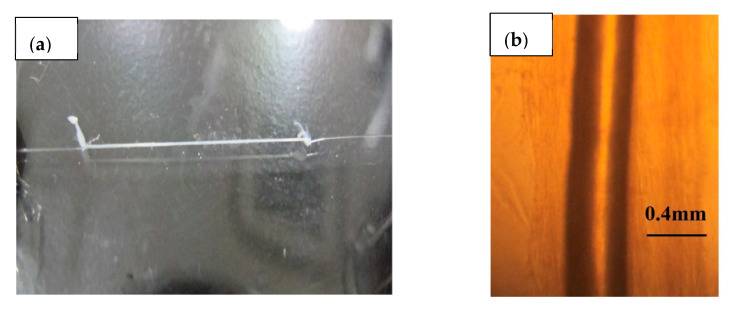
Photograph of the extracted axon: (**a**) overview and (**b**) partial view under the microscope.

**Figure 2:**
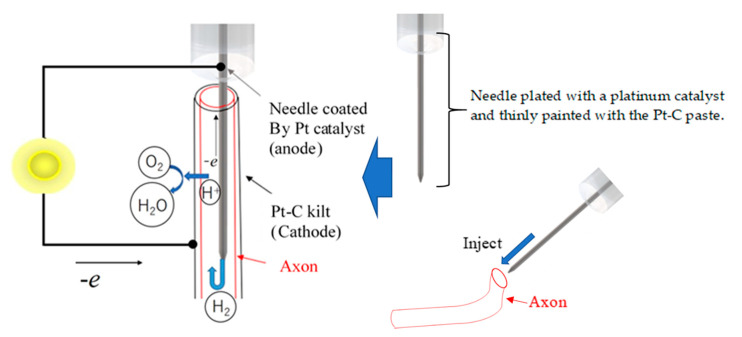
Schematic diagram of the fuel cell based on the axon electrolyte.

Specimens of axon were extracted from squids (*Uroteuthis edulis*) and used as ion channel membranes. In the natural environment, squid inhabit a water temperature of 15–20 °C and a water depth of 60–100 m. The squid caught in the sea near Hakodate was transported alive to the laboratory at room temperature and decapitated before the experiment. The collected axons were handled at room temperature (around 20 °C) during the experiment. The axons in the mantle were immediately separated from the stellate ganglion and washed with distilled water after removing the internal matrix. Figure 1a,b shows the extracted axons. As shown in Figure 1a, the axon was extracted cleanly and its length was enough to fabricate the fuel cell electrolyte. As shown in Figure 1b, the axon was translucent and had a tubular structure. The axon was approximately 0.020 mm in thickness and approximately 0.45 mm in diameter. The collected axons were stored at 4 °C in an ice bath. Figure 2 shows a schematic diagram of the fuel cell using the axon as the electrolyte. As shown in Figure 2, the needle was inserted inside the axon without breaking the axon and used as the anode electrode of the fuel cell. The stainless steel needle is 0.15 mm in diameter and 5.0 mm in length. In order to use the needle as the fuel cell electrode, the surface of the needle was plated with a platinum catalyst, and the Pt-C paste was thinly painted not only to adhere to the inner wall of the axon and needle but also to facilitate the penetration of the hydrogen gas to the inner wall of the axon. The hydrogen gas as fuel was provided into the inside of the axon through the needle. The cathode electrode was fabricated by wrapping a Pt-C quilt around the outer surface of the axon. The oxygen required for power generation was supplied from the air.

**Figure 3:**
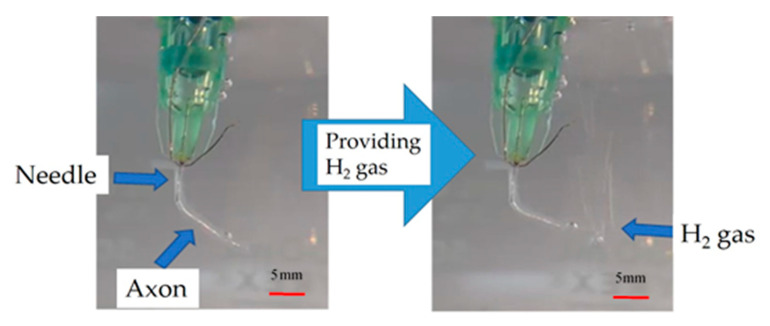
Injection of a needle inserted into the axon.

Figure 3 shows a photograph in which hydrogen gas was supplied inside the axon by inserting the needle into the axon. As shown in Figure 3, when the hydrogen gas was provided into the axon from the needle, the hydrogen gas passed through without leaking from the axon’s surface. This fact implies that the axon can be used as a fuel cell electrolyte, considering that blocking the fuel gas from the inside to the outside of electrolytes does not decrease the power of the fuel cell.

### 2.2. Experimental Procedures of the Fuel Cell Characteristics and Proton Conductivity

The current density versus cell voltage characteristics of the fuel cell was measured at room temperature with a low-noise voltmeter (Keithley2010, Keithley Instruments, Cleveland, OH, USA), a digital multimeter (Keithley2000, Keithley Instruments), and a measurement-control computer.

Proton conductivity measurement was carried out using a precision LCR meter (E4980A, Agilent Technologies Inc., Santa Clara, CA, USA) in the frequency range of 100 Hz to 1 MHz at room temperature. The relative humidity was controlled by a humidified gas flow system (Auto PEM, Toyo Corporation, Tokyo, Japan). For proton conductivity measurement, the admittance, which consists of the conductance and susceptance, was measured. The DC proton conductivity *σ*_0_ and static dielectric constant *ε*_s_ of the ion channel were obtained from analyzing the frequency dependences of conductance and susceptance.

## 3. Results

**Figure 4:**
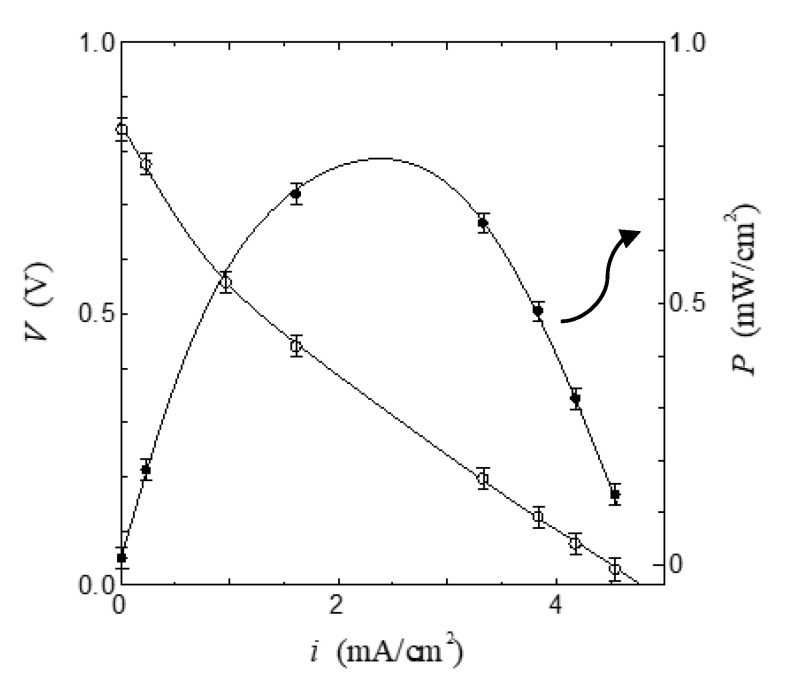
Relationship between current density *i* and cell voltage *V* in the fuel cell based on the ion channel membrane. Solid lines are a guide to the eyes.

**Figure 5:**
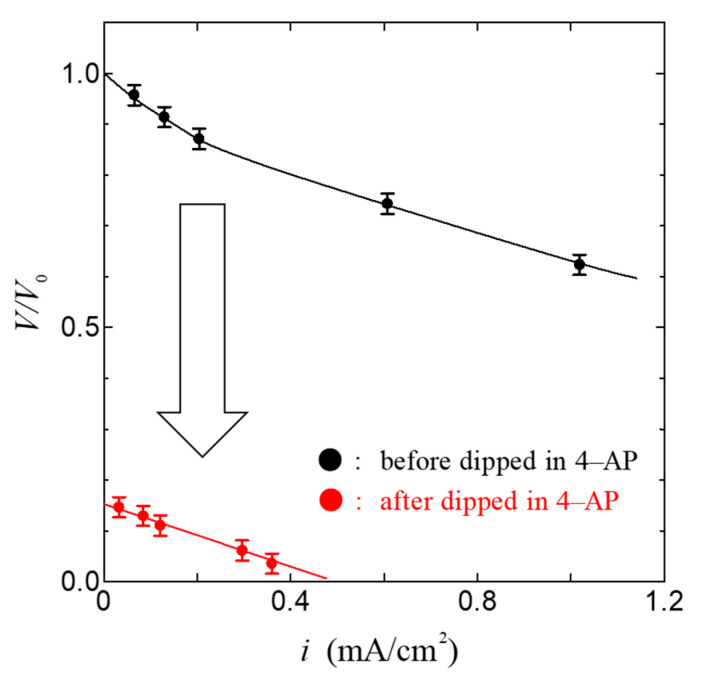
Change in *i*–*V* characteristics by introducing 4-aminopyridine (4-AP) conditions. The Solid line is a guide to the eyes.

Figure 4 shows the relationship between the current density *i* and the cell voltage *V* in the fuel cell using the ion channel electrolyte at 100% relative humidity. As shown in Figure 4, the open-circuit voltage of this fuel cell was 0.84 V and the maximum power density was 0.78 mW/cm^2^. The value of power density in the fuel cell based on ion channel electrolyte is higher than that in other biopolymers such as chitin, chitosan, and collagen membranes [28,29]. This result indicates that the membrane, including the ion channel, is a superior proton conductor and could become a superior electrolyte of fuel cells. In order to confirm the role of the ion channel in the operation of the fuel cell, we have measured the *i*–*V* characteristics using the ion channel blocker 4-aminopyridine(4-AP, Nacalai Tesque, Tokyo, Japan) [37,38,42,43] and compared them with the *i*–*V* characteristics using the ion channel electrolyte without the channel blocker. Figure 5 shows the relationship between the current density *i* and the normalized cell voltage *V*/*V*_0_ of the fuel cell dipped in 4-aminopyridine at 100% relative humidity. Here, *V*_0_ was obtained from the open-circuit voltage of the ion channel electrolyte fuel cell before being dipped in 4-aminopyridine; the error bar in Figure 4 and Figure 5 is mainly related to individual differences in the axon specimens.

As shown in Figure 5, *V*/*V*_0_ and *i* decreased extremely. These results indicate that the lowering of the current density is caused by the blocking of proton transfer via the ion channel due to the ion channel blocker. Therefore, it is deduced that the ion channel plays an important role in the power generation of the fuel cell.

In order to investigate the proton conductivity of the ion channel electrolyte, we have measured the relative humidity dependence of proton conductivity in the ion channel electrolyte, because the proton conductivity of the ion channel electrolyte is a key factor for the determination of the power generation by a fuel cell.

**Figure 6:**
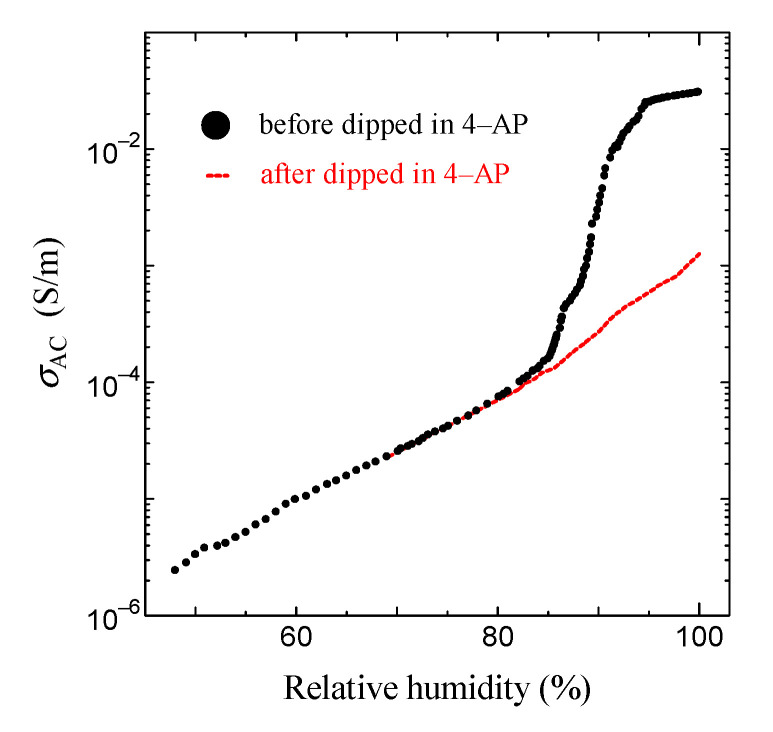
Relative humidity dependence of AC proton conductivity (*σ*_AC_). The solid black circle is the result before being dipped in 4–AP; the red circle is the result after being dipped in 4-AP.

**Figure 7:**
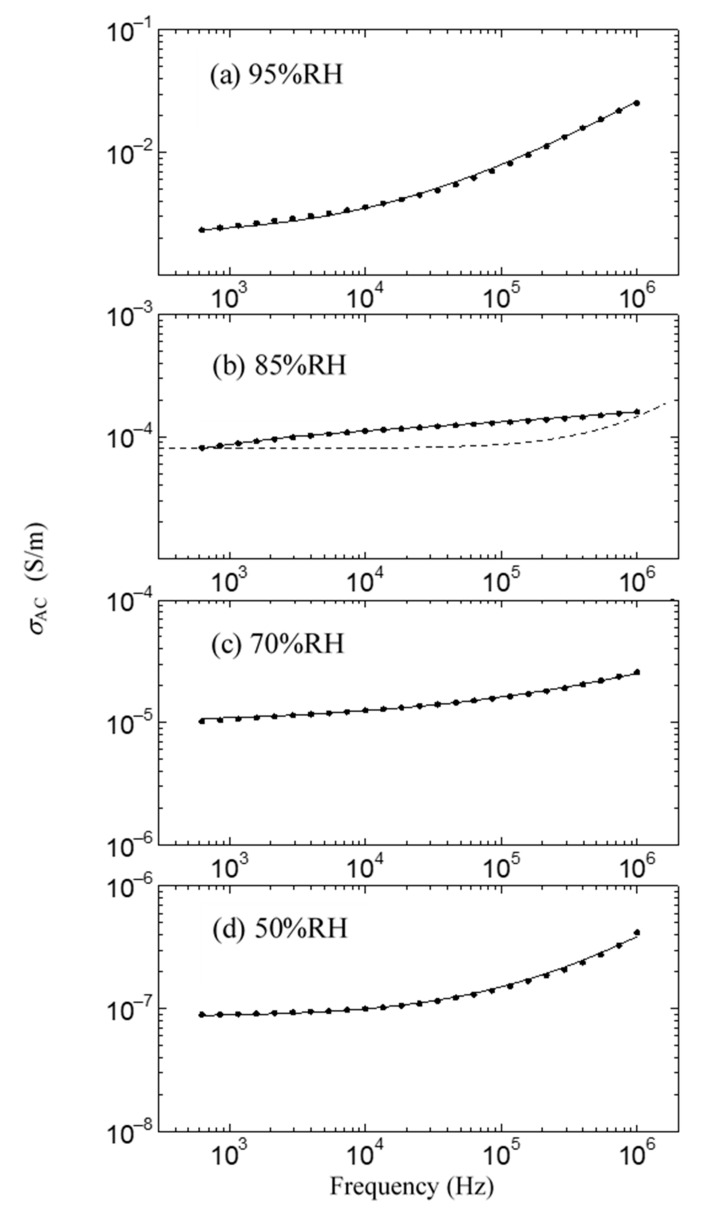
Frequency dependence of AC proton conductivity *σ*_AC_ at various relative humidity levels. (**a**) 95% RH; (**b**) 85% RH; (**c**) 70% RH; (**d**) 50% RH. Solid lines show the results calculated by Equation (1).

This result is shown in Figure 6. As shown in the solid black circle in Figure 6, the proton conductivity of the ion channel electrolyte increases with the increase in humidity, drastically increases at around 85% relative humidity (RH), and then becomes 2.6 × 10^−2^ S/m at 96% RH. On the other hand, above 85% RH, we cannot observe any drastic increase in proton conductivity after it has been dipped in the ion channel blocker 4-AP. It is well known that hydration is essential for the functional expression of proteins. Therefore, these results indicate that the drastic increase in proton conductivity observed above 85% RH will be caused by the activation of ion channels by hydration [44]. Figure 7 shows the frequency dependence of the AC proton conductivity of the ion channel electrolyte observed at various relative humidity levels. As shown in Figure 7, *σ*_AC_ increases with the increase in relative humidity, as seen in Figure 6. Moreover, the shape of the frequency dependence of *σ*_AC_ changes with the relative humidity. This result is a characteristic feature of the ion channel electrolyte and is discussed in the next section.

## 4. Discussion

The present study aims to report the fabrication of a fuel cell using the ion channel electrolyte and show its properties from the viewpoint of proton conductivity. As described above, the ion channel membrane is a superior proton conductor at 100% RH and we can fabricate a fuel cell by using the ion channel electrolyte of the fuel cell. This result is one of our aims. Next, we would like to discuss proton conductivity in the ion channel electrolyte.

As shown in Figure 6 and Figure 7, it is noted that the frequency dependence of *σ*_AC_ changes with the relative humidity and shows convex upward dependence around 1 kHz at 85% RH, where a drastic increase in proton conductivity is observed. In Figure 7, the proton conductivity *σ*_AC_ of the solid circle was obtained from the simple equation *σ*_AC_ = *σ*_0_ + *ωε*_0_*ε*”, which is obtained assuming that the ion channel electrolyte is expressed by the simple parallel equivalent circuit of the capacitance and resistance. Here, *ω* is the angular frequency, and *ε*_0_ and *ε*” are the dielectric constant in a vacuum and the imaginary part of the complex dielectric constant, respectively. The symbol *σ*_0_ is DC proton conductivity, which is obtained from the real part of the measured impedance. Therefore, in the case that the ion channel electrolyte is described as this simple parallel equivalent circuit, *σ*_AC_ is proportional to *ω* such as the dotted line shown in Figure 7b, because *σ*_0_ and ε” become constant in the exact equivalent circuit. However, as seen in Figure 7b, the obtained frequency dependence of *σ*_AC_ does not obey the dotted line but shows convex upward dependence. This frequency dependence is observed in the case that the component of the dielectric dispersion is included in the AC proton conductivity [27]. That is, the component of dielectric dispersion remarkably appears above 85% RH. Therefore, it is necessary to consider the AC proton conductivity, including the dielectric dispersion.

The following equation describes the AC proton conductivity, including the dielectric dispersion:

(1) σAC=σ0−Im[ωε0ε∞+ωε0(εs−ε∞)1+(jωτ)β]=σ0+ωε0(εs−ε∞)(ωτ)βsin(π2β)(1+(ωτ)βcos(π2β))2+((ωτ)βsin(π2))2

where *ε*_s_ and *ε*_∞_ are the static and unrelaxed dielectric constants, respectively; *τ* is the relaxation time for the dielectric dispersion. The symbol of *β* indicates the degree of multi-dispersion.

**Figure 8:**
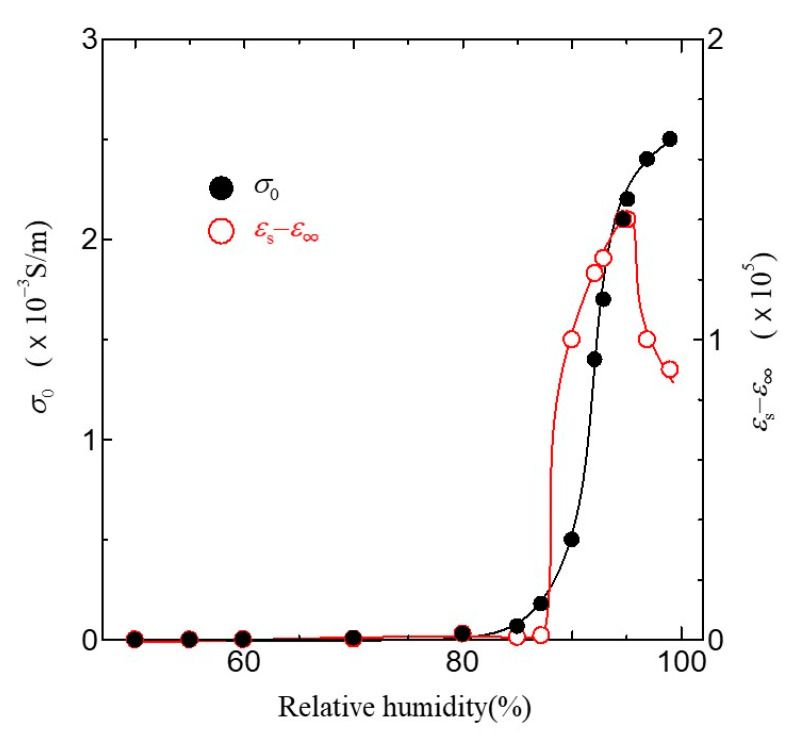
Relative humidity dependence of σ_0_ and *ε*_s_*−ε*_∞._ Solid lines are a guide to the eyes.

**Figure 9:**
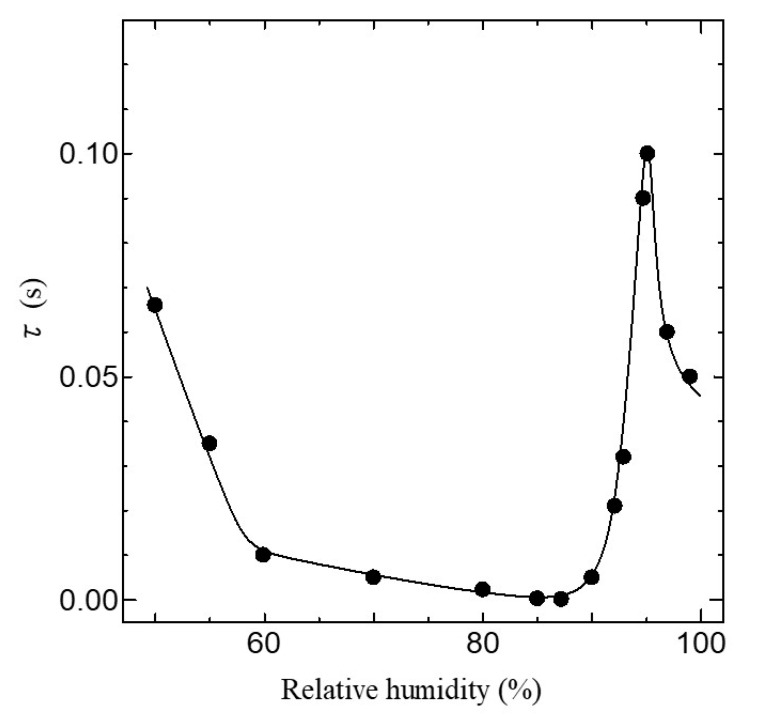
Relative humidity dependence of *τ.* The solid line is a guide to the eyes.

The dielectric dispersion becomes mono-dispersion when *β* = 0. Using Equation (1), we can calculate the frequency dependence of *σ*_AC_ in the ion channel membrane. The calculated results are shown as the solid line in Figure 7. The values of the parameter used in the fitting are listed in Table 1. As shown in Figure 7, it is evident that the calculated results are in excellent agreement with the experimental ones in all relative humidity conditions. Figure 8 shows the relative humidity dependences of DC proton conductivity *σ*_0_ and the dielectric constant *ε*_s_−*ε*_∞_ obtained from Equation (1). As shown in Figure 8, *σ*_0_ drastically increases above around 85% RH. The drastic increase in proton conductivity above around 85% RH is caused by the existence of the ion channel, as shown in Figure 6. Therefore, it is deduced that the increase in *σ*_0_ above around 85%RH results from the activation of proton transport by the hydration of ion channels. The dielectric constant *ε*_s_−*ε*_∞_ also increases drastically from at around 85% RH and takes the maximum value at around 96% RH. It is noteworthy that *σ*_0_ and *ε*_s_−*ε*_∞_ drastically increase at the same relative humidity of 85%. These results indicate that the dielectric anomaly observed at around 85% RH appears from the same cause as the appearance of proton conductivity. That is, the activation of ion channels by hydration leads to an increase in *ε*_s_−*ε*_∞_ at around 85% RH. The increase in humidity yields an increase in water molecules in the axon. The water molecules have an electric dipole moment. Therefore, the motion of the dipole moment of water molecules reflects the value of *ε*_s_−*ε*_∞_. The motion of the dipole moment of water molecules above around 85% RH can be described from the behavior of the dielectric relaxation time. Figure 9 shows the humidity dependence of the dielectric relaxation time *τ*. As shown in Figure 9, *τ* begins to increase at around 85% RH and takes the maximum value at 96% RH. It is also noted that the value of *τ* is as long as 10^−4^ s or more. Considering that the dielectric relaxation time of free water is shorter than one ns, this result indicates that the observed dielectric relaxation results from the water molecule clusters bonding with the axon, similar to what is observed in the water bridges of humidified DNA [26]. The value of *τ* strongly depends on the size of the water molecule clusters bonded with the axon. When the correlation between water molecules becomes strong and the large water molecule clusters are formed, *τ* becomes long. On the contrary, *τ* becomes short with the fractionalization of water molecule clusters. It is deduced that the increase in *τ* above around 85% RH results from the formation of large clusters of water molecules with the increase in water molecules, and the decrease in *τ* above 96% RH is caused by the appearance of the fractionalization of water molecule clusters. It is known that when the ion channel becomes active, the water molecule separates from the cluster of water molecules and leads to ion transport by realizing the coupling between ions and the water molecule [45,46]. That is, the activation of the ion channel yields the fractionalization of water molecule clusters. This is consistent with the decrease in *τ* observed above 96% RH. It is well known that many functional proteins, including ion channels, have a strong relationship between hydration and structure, and commonly have an activated structure in water. In addition, proton conductivity above 96% RH is closely related to the ion channel. It is deduced that the structural changes of ion channels due to hydration lead to the fractionation of water clusters above 96% RH. According to L.J. Mullins [36], the ion exchange occurs in pores (ion channels) in axons. Considering these results, it is speculated that the structural change of the ion channel at 96% RH causes the fractionation of water molecule clusters, and the ion channel with the activated structure leads to proton conduction.

## 5. Conclusions

In the present work, we have fabricated a fuel cell using the electrolyte of the ion channel membrane extracted from the squid axon. The application to the fuel cell of the ion channel membrane is the first attempt. The fuel cell fabricated using the ion channel membrane showed a power density of 0.78 mW/cm^2^. Moreover, the significant reduction in power due to the use of a channel blocker (4-AP) indicates that the power is yielded by proton conduction of the ion channels in the axon. Furthermore, the electrical properties of the ion channel electrolyte were also investigated. As a result, it was found that the ion channel electrolyte has high proton conductivity above 96% RH. In addition, the analysis of dielectric properties indicated that the fractionalization of water molecule clusters is realized above 96% RH, where high proton conductivity is obtained. This result is consistent with other results in which the ion channel transports water molecules together with ions. These results indicate that the fuel cell using the squid axon could become a new fuel cell using the function of the ion channel above 96% RH. Moreover, we have found that the ion channel operates as the electrolyte of a fuel cell, although the proton conductivity of the electrolyte using the ion channel is same as or low compared with the biopolymer electrolyte of chitin. This result will yield new developments such as artificial ion channel membranes with high-speed proton conductivity, in which ion channels are selectively implanted.

## Figures and Tables

**Table 1:** Values of DC proton conductivity (*σ*_0_), the dielectric constant (*ε*_s_−*ε*_∞_), dielectric relaxation time (*τ*), and degree of multi-dispersion (*β*) used in the fitting of the observed frequency dependence of *σ*_AC_ at various relative humidity.

RH (%)	*σ*_0_ (S/m)	*ε*_s_−*ε*_∞_	*τ* (s)	*β*
95	2.10 × 10^−3^	1.40 × 10^5^	9.00 × 10^−2^	0.395
85	6.80 × 10^−5^	9.00 × 10^2^	2.55 × 10^−4^	0.850
70	9.20 × 10^−6^	3.00 × 10^2^	5.00 × 10^−3^	0.660
50	4.80 × 10^−7^	1.10 × 10^2^	6.60 × 10^−2^	0.574
